# Clarification

**Published:** 2009-05

On p. A155 of the April Focus article [*Environ Health Perspect* 117:A150–A156 (2009)], a statement reads, “The primary global impact of oil sands comes through the release of greenhouse gases created when about 800 million cubic feet of natural gas (approximately 10% of Canada’s total natural gas consumption) is burned daily to create heat for extraction and upgrading, says Stringham.” To clarify: This statement refers to the global impact of oil sands *production*. As noted in the preceding paragraph of the article, most of the greenhouse gas emissions related to oil sands overall come from burning the fuel derived from these reserves.

